# Association of Preoperative Functional Status With Short-Term Major Adverse Outcomes After Cardiac Surgery

**DOI:** 10.7759/cureus.80586

**Published:** 2025-03-14

**Authors:** Barbara Chiu, Julio E Sanchez Gonzalez, Isabel Diaz, Pura Rodriguez de la Vega, Rupa Seetharamaiah, Georgeta Vaidean

**Affiliations:** 1 Department of Medical Education, Florida International University, Herbert Wertheim College of Medicine, Miami, USA; 2 Department of Medical and Population Health Sciences Research, Florida International University, Herbert Wertheim College of Medicine, Miami, USA; 3 Department of Surgery, Florida International University, Herbert Wertheim College of Medicine, Miami, USA; 4 Department of Surgery, Baptist Hospital of Miami, Miami, USA

**Keywords:** adverse outcomes, cardiac surgery, cardiovascular disease, dependency, functional status, morbidity, mortality, nsqip, risk factors

## Abstract

Introduction

Cardiac surgery plays a crucial role in treating a wide range of cardiovascular conditions, offering life-saving interventions for patients with diseases such as coronary artery disease, heart valve disorders, and heart failure. However, these procedures are not without significant risks, including complications such as stroke, acute kidney injury, respiratory failure, and infections. It is important to not only recognize the potential complications associated with these procedures but also identify high-risk patients early in the treatment process. With the aging population and the increasing burden of comorbidities, a growing number of patients are likely to present with suboptimal functional status prior to cardiac surgery. By incorporating functional status into preoperative evaluations, healthcare providers can improve patient selection, enhance perioperative care, and improve outcomes in this high-risk patient population. Therefore, this study aims to investigate whether preoperative dependent functional status is associated with an increased risk of postoperative major adverse outcomes in patients undergoing cardiac surgery.

Methods

We performed a retrospective cohort analysis on adult cardiac surgery patients based on the American College of Surgeons National Surgical Quality Improvement Program (ACS NSQIP) 2011-2021 database. We compared a primary composite outcome consisting of post-surgery outcomes between independent and partially/totally dependent patients. The primary outcome was defined as experiencing any of the following adverse events: superficial incisional/deep incisional/organ space surgical site infection, death within 30 days post-operation, stroke/cerebral vascular accident (CVA), cardiac arrest requiring cardiopulmonary resuscitation (CPR), myocardial infarction, pulmonary embolism (PE), deep vein thrombosis (DVT)/thrombophlebitis, progressive renal insufficiency, ventilator use for more than 48 hours post-operation, unplanned intubation or reoperation, sepsis, septic shock, and pneumonia. Confounding variables were age, gender, race, emergency case, comorbidities, and baseline laboratory markers. We used multivariable logistic regression analysis to obtain adjusted odds ratio (OR) and 95% confidence intervals (CIs).

Results

Of the 42,917 patients included in the study, 30.6% were female and 69.4% were male, with 46.5% of the group being 65-79 years old. The prevalence of dependent status was 2.6%. Compared to independent patients, those who were dependent had a higher incidence of the primary outcome (35.68% vs. 20.93%), yielding a crude OR of 2.09 (95% CI 1.85-2.37). The association remained significant: OR of 1.21 (95% CI 1.04-1.41) after adjustment for age, gender, race, body mass index (BMI), emergency case, and other comorbidities such as diabetes, hypertension, heart failure, preoperative blood transfusion or sepsis, and laboratory markers.

Conclusion

Patients with preoperative dependent functional status were found to have a significantly greater risk of complications after cardiac surgery, even after adjusting for demographics, comorbidities, laboratory markers, and perioperative characteristics. Further investigation is needed to explore the development and clinical application of a predictive tool that includes functional status, which could help identify high-risk patients and facilitate timely interventions such as prehabilitation programs to enhance functional capacity.

## Introduction

Despite progress in surgical techniques and perioperative management, complication rates in patients undergoing cardiac surgery remain a significant concern. For instance, in a study that analyzed 2,477 patients who underwent cardiac procedures, 14.8% of patients experienced at least one postoperative complication, with a mortality rate of 5.2%, and 4.1% experienced multiple postoperative complications, with a mortality rate of 41% [1]. The most common complications identified included stroke, prolonged intubation, renal failure, unplanned reoperation, and deep sternal wound infections. Other studies have identified postoperative cardiac arrhythmia as a major complication following cardiac surgery [2]. With the rising burden of cardiovascular diseases and the substantial number of cardiac procedures performed each year, a better understanding of postoperative complications will remain critical for patient risk stratification, perioperative management, and shared decision-making [3,4].

While previous investigations have explored factors such as age, gender, body mass index (BMI), operation time, tobacco use, and laboratory values to assess the risk of adverse events in patients undergoing cardiac surgery, the impact of functional status has received less attention [5]. Functional status refers to a patient's capacity to engage in activities of daily living (ADLs), such as bathing and feeding themselves. Patients who do not require assistance from another person to perform these activities are classified as independent, whereas those who need partial or total assistance with ADLs are classified as dependent [6]. The predictive capacity of functional status has been extensively assessed in studies focusing on non-cardiac surgeries [7,8]. For example, a study that aimed to predict the development of complications after spinal surgery found that dependent patients were 2.1 times more likely to experience complications compared to independent patients [7]. Researchers suggested this may be due to frailty, an increased vulnerability to stressors often associated with comorbidities, though functional dependence itself remained a key risk factor for complications [7]. Building upon this evidence and aiming to address the gap in the existing literature on this topic, our study aims to investigate the association between preoperative functional status and short-term major postoperative complications in cardiac surgery.

## Materials and methods

Study design and population

We performed a retrospective cohort study with secondary data analysis of patient data recorded in the American College of Surgeons National Surgical Quality Improvement Program (ACS NSQIP) database during 2011-2021. The database is a nationally validated, outcomes-based program designed to measure and improve the quality of surgical care [9]. Institutional Review Board approval was not required as this study utilized de-identified data.

There were 43,176 patients over the age of 18 who underwent cardiac surgery between the years 2011 and 2021. Cardiac surgery was defined by the NSQIP “surgical specialty” code, which characterizes the principal operative procedure based on the surgeon’s self-declared specialty. Patients were excluded if they had an unknown preoperative functional status and missing data in any of the outcomes listed in the primary composite outcome. The final cohort included 42,917 patients. 

The exposure in our study was preoperative functional status. NSQIP defined functional status as independent, partially independent, totally dependent, and unknown; we combined partially dependent and totally dependent (referred to as “dependent”) and contrasted this category with the independent status. The primary outcome was a composite of adverse events including superficial incisional/deep incisional/organ space surgical site infection, death within 30 days post-operation, stroke/cerebral vascular accident (CVA), cardiac arrest requiring CPR, myocardial infarction, pulmonary embolism, deep vein thrombosis (DVT)/thrombophlebitis, progressive renal insufficiency, ventilator use for more than 48 hours post-operation, unplanned intubation, sepsis, septic shock, pneumonia, and unplanned reoperation. 

Patient demographics (age, gender, race, and ethnicity) and clinical baseline characteristics considered were history of diabetes mellitus, current smoker within one year, dyspnea, history of severe chronic obstructive pulmonary disease (COPD), heart failure within 30 days before surgery, hypertension requiring medication, preoperative acute renal failure, preoperative currently on dialysis, disseminated cancer, immunosuppressive therapy, malnourishment, bleeding disorders, preoperative transfusion greater than one unit of whole/packed red blood cells (RBCs) in 72 hours prior to surgery, open wound/wound infection, and systemic sepsis. Patient preoperative laboratory markers/biomarkers consisted of creatinine, serum albumin, total bilirubin, white blood cell (WBC), hematocrit (Hct), platelet count, partial thromboplastin time (PTT), international normalized ratio (INR) of prothrombin, and prothrombin time. Operative and perioperative characteristics included total operation time, principal operative procedure current procedural terminology (CPT) code description, elective surgery, emergency case, ventilator dependent, length of total hospital stay, and discharge destination. 

Statistical analysis

The distribution of baseline patient characteristics was compared between independent and dependent functional status groups using the chi-square test. The incidence of the primary composite outcome was compared by patient characteristics in each category. Laboratory values were categorized based on literature-based cutoffs.

Binary logistic multivariable regression was used to calculate the odds of developing the primary outcome as a function of functional status. Candidate covariates for statistical modeling were selected based on clinical judgment and retained in the model if they changed the main estimate by more than 10%. The Stata software version 15 (2017) (StataCorp LLC, College Station, TX) was used for all statistical analyses [10]. A p-value of less than 0.05 was considered statistically significant.

## Results

Our study included 42,917 patients; of that, 97.3% of the patients were classified as independent and 2.6% of the patients were classified as dependent.

Table 1 compares the baseline characteristics of the participants in the dependent functional status group vs. the independent functional status group. The dependent status group had a significantly greater proportion of patients who were older than 80 years, female, and non-White (p < 0.001 for all comparisons). There was also a higher percentage of patients classified as underweight (1.66% vs. 0.96%) and patients with obesity (41.46% vs. 40.90%). In the dependent group, there was a lower percentage of overweight participants (30.56% vs. 36.5%). The dependent group had greater prevalence of comorbidities except for current smoking status. Dependent patients were also more likely than independent patients to be emergency operative cases (14.19% vs. 8.51%) and be ventilator-dependent (7.75% vs. 1.01%) prior to surgery. Lastly, there was a greater percentage of abnormal laboratory markers (creatinine, albumin, WBC, Hct) in the dependent group.

**Table 1:** Baseline characteristics of patients undergoing  cardiac surgery by functional status (NSQIP 2011-2021) ^1^Dependent functional status was classified as totally or partially dependent. COPD = chronic obstructive pulmonary disease, CHF = congestive heart failure, HTN = hypertension, NSQIP = National Surgical Quality Improvement Program, SIRS = systemic inflammatory response syndrome, WBC = white blood cell

Characteristics	Preoperative functional status		p-value
	Independent (N = 41782)	Dependent^1^ (N = 1135)	
	N (%)	N (%)	
Demographics			
Age (years)			<0.001
<50	4326 (10.35)	88 (7.75)	-
50-64	13491 (32.39)	322 (28.37)	
65-79	19400 (46.43)	517 (45.55)	
≥80	4565 (10.93)	208 (18.33)	
Gender			<0.001
Female	12634 (30.24)	478 (42.11)	-
BMI			<0.001
Underweight (<18)	395 (0.96)	18 (1.66)	-
Normal (18-25)	8984 (21.75)	285 (26.32)	
Overweight (25-29)	15033 (36.5)	331 (30.56)	
Obese (30+)	16892 (40.9)	449 (41.46)	
Race			<0.001
White	24754 (87.02)	724 (78.61)	-
Black	2205 (7.75)	115 (12.49)	
Other	1487 (5.23)	82 (8.9)	
Ethnicity			<0.001
Hispanic	2831 (9.93)	165 (17.48)	-
Comorbidities			
Diabetes mellitus	13117 (31.39)	496 (43.7)	<0.001
Dyspnea	12953 (34.66)	490 (48.13)	<0.001
Current smoker	8257 (19.76)	217 (19.12)	0.591
COPD	3429 (8.21)	173 (15.25)	<0.001
CHF	5084 (12.17)	330 (29.07)	<0.001
HTN	30809 (73.74)	903 (79.56)	<0.001
Dialysis	1016 (2.43)	134 (11.81)	<0.001
Cancer	361 (0.86)	25 (2.2)	<0.001
Steroid use	1373 (3.29)	82 (7.22)	<0.001
Bleeding disorder	4715 (11.28)	256 (22.56)	<0.001
Blood transfusion	1054 (2.52)	105 (9.25)	<0.001
Wound infections	627 (1.68)	121 (11.89)	<0.001
Systemic inflammation			<0.001
None	40.46 (95.85)	974 (85.81)	-
SIRS	1328 (3.18)	105 (9.25)	
Sepsis/shock	408 (0.98)	56 (4.93)	
Perioperative and operative characteristics			
Emergency case	3554 (8.51)	161 (14.19)	<0.001
Ventilator dependent	423 (1.01)	88 (7.75)	<0.001
Baseline laboratory markers (preoperative)			
Creatinine (mg/dL)	-	-	<0.001
>1.5	4294 (10.49)	294 (26.13)	-
Albumin (g/dL)	-	-	<0.001
>3.5	24839 (73.64)	399 (44.04)	-
WBC (10^9^/L)	-	-	<0.001
<4 or >10	7229 (17.62)	328 (29.26)	-
Hematocrit (%)	-	-	<0.001
<40	20343 (49.59)	878 (78.11)	-

The incidence of the 30-day postoperative complications included in the primary composite outcome are listed in Table 2. The most common complications included unplanned reoperation (6.80%), being on a ventilator >48 hours (5.80%), pneumonia (5.60%), and unplanned intubation (3.30%). Demographic factors associated with higher incidence of complications include age >80 years (24.83%), females (23.02%), BMI <18 (27.36%), and Blacks (26.38%). There was no significance between those of Hispanic vs. non-Hispanic ethnicity on the incidence of complications. Several comorbidities were associated with a higher incidence of complications, especially sepsis/shock (68.75%), systemic inflammatory response syndrome (SIRS) (43.13%), blood transfusion (42.54%), dialysis (39.48%), congestive heart failure (CHF) (35.7%), and wound infections (34.89%). Moreover, complications included in the composite outcome were observed in 75.73% of participants who were ventilator-dependent and in 37.28% of emergency cases. Abnormal values of creatinine, albumin, WBC, and Hct were significantly associated with postoperative complications.

**Table 2:** Incidence of 30-day postoperative complications included in the primary composite outcome DVT = deep venous thrombosis, MI = myocardial infarction, PE = pulmonary embolism, SSI = surgical site infection

Primary outcome	Frequency N (%)
Operative	
Superficial incisional SSI	1281 (3)
Deep incisional SSI	196 (0.5)
Organ space SSI	204 (0.5)
Systemic	
Death within 30 days	1283 (3)
Stroke	782 (1.8)
Cardiac arrest	1023 (2.4)
MI	346 (0.8)
PE	232 (0.5)
DVT	488 (1.1)
Renal	856 (2)
Ventilator	2471 (5.8)
Unplanned intubation	1417 (3.3)
Sepsis	629 (1.5)
Septic shock	542 (1.3)
Pneumonia	2400 (5.6)
Other	
Unplanned reoperation	2917 (6.8)

The incidence of the primary composite outcome by patient characteristics is presented in Table 3. The proportion of participants who experienced the primary outcome was greater in those with dependent functional status (35.68%) compared to those who were independent (20.93%). In unadjusted analysis, dependent patients were 2.09 times more likely to experience a complication compared to independent patients (2.09 OR, 95% CI 1.85-2.37). After multivariable adjustment, the adjusted OR was 1.21 (95% CI 1.04-1.41). The confounders that we adjusted for in our model included age, gender, BMI, CHF, dialysis, bleeding disorder, blood transfusion, SIRS, sepsis/shock, perioperative ventilator dependence, perioperative creatinine, perioperative albumin, perioperative WBC, and perioperative Hct (Table 4).

**Table 3:** Incidence of primary composite outcome by patient characteristics ^1^As defined in Table 2 COPD = chronic obstructive pulmonary disease, CHF = congestive heart failure, HTN = hypertension, SIRS = systemic inflammatory response syndrome, WBC = white blood cell

Characteristics	Primary outcome^1^	
	Yes (N = 9152)	No (N = 33765)
	N (%)	N (%)
Functional status		
Independent	8747 (20.93)	33035 (79.07)
Dependent	405 (35.68)	730 (64.32)
Demographics		
Age (years)		
<50	884 (20.02)	3530 (79.97)
50-64	2722 (19.71)	11091 (80.29)
65-79	4361 (21.9)	15556 (78.1)
≥80	1185 (24.83)	3588 (75.17)
Gender		
Male	6131 (20.57)	23671 (79.43)
Female	3019 (23.02)	10093 (76.98)
BMI		
Underweight (<18)	113 (27.36)	300 (72.64)
Normal (18-25)	2044 (22.05)	7225 (77.95)
Overweight (25-29)	2942 (19.15)	12422 (80.85)
Obese (30+)	3883 (22.39)	13458 (77.61)
Race		
White	5248 (20.6)	20230 (79.4)
Black	612 (26.38)	1708 (73.62)
Other	306 (19.5)	1263 (80.5)
Ethnicity		
Hispanic	5376 (20.33)	21074 (79.67)
Non-Hispanic	647 (21.6)	2349 (78.4)
Comorbidities		
Diabetes mellitus	3200 (23.51)	10413 (76.49)
Dyspnea	3317 (24.67)	10126 (75.33)
Current smoker	1968 (23.22)	6506 (76.78)
COPD	1077 (29.9)	2525 (70.1)
CHF	1933 (35.7)	3481 (64.3)
HTN	7033 (22.18)	24679 (77.82)
Dialysis	454 (39.48)	696 (60.52)
Cancer	118 (30.57)	268 (69.43)
Steroid use	436 (29.97)	1019 (70.03)
Bleeding disorder	1457 (29.31)	3514 (70.69)
Blood transfusion	493 (42.54)	666 (57.46)
Wound infections	261 (34.89)	487 (65.11)
Systemic inflammation		
None	8215 (20.03)	32805 (79.97)
SIRS	618 (43.13)	815 (56.87)
Sepsis/shock	319 (68.75)	145 (31.25)
Peri-operative and operative characteristics		
Emergency case	1385 (37.28)	2330 (62.72)
Ventilator dependent	387 (75.73)	124 (24.27)
Baseline laboratory markers (preoperative)		
Creatinine (mg/dL)		
<1.5	7404 (19.75)	30076 (80.25)
>1.5	1641 (35.77)	2947 (64.23)
Albumin (g/dL)		
>3.5	4677 (18.53)	20561 (81.47)
≤3.5	3034 (32.38)	6365 (67.72)
WBC (10^9^/L)		
4 to 10	6670 (19.29)	27913 (80.71)
<4 to >10	2369 (31.35)	5188 (68.85)
Hematocrit (%)		
≥40	3643 (17.41)	17283 (82.59)
<40	5401 (25.45)	15820 (74.55)

**Table 4:** Adjusted and unadjusted associations between patient characteristics and primary outcome COPD = chronic obstructive pulmonary disease, CHF = congestive heart failure, HTN = hypertension, SIRS = systemic inflammatory response syndrome, WBC = white blood cell

Characteristics	Unadjusted		Adjusted	
	OR (95% CI)	p-value	OR (95% CI)	p-value
Functional status				
Independent	Reference	-	-	-
Dependent	2.09 (1.85-2.37)	<0.001	1.21 (1.04-1.41)	0.015
Demographics				
Age (years)				
<50	Reference	-	-	-
50-64	0.98 (0.90-1.07)	0.641	1.01 (0.91-1.11)	0.905
65-79	1.12 (1.03-1.21)	0.006	1.17 (1.06-1.29)	0.001
≥80	1.32 (1.19-1.46)	<0.001	1.34 (1.19-1.51)	<0.001
Gender				
Male	Reference	-	-	-
Female	1.15 (1.09-1.21)	<0.001	1.09 (1.03-1.16)	0.005
BMI				
Underweight (<18)	1.33 (1.07-1.66)	0.011	1.14 (0.88-1.48)	0.305
Normal (18-25)	Reference	-	-	-
Overweight (25-29)	0.84 (0.79-0.89)	<0.001	0.92 (0.86-0.99)	0.031
Obese (>30)	1.02 (0.96-1.08)	0.525	1.12 (1.05-1.20)	0.001
Race				
White	Reference	-	-	-
Black	1.38 (1.25-1.52)	<0.001	-	-
Other	0.93 (0.82-1.06)	0.297	-	-
Ethnicity				
Hispanic	1.08 (0.98-1.18)	0.102	-	-
Non-Hispanic	Reference	-	-	-
Comorbidities				
Diabetes mellitus	1.21 (1.15-1.27)	<0.001	-	-
Dyspnea	1.39 (1.32-1.46)	<0.001	-	-
Current smoker	1.15 (1.08-1.21)	<0.001	-	-
COPD	1.65 (1.53-1.78)	<0.001	-	-
CHF	2.33 (2.19-2.48)	<0.001	1.66 (1.55-1.78)	<0.001
HTN	1.22 (1.16-1.29)	<0.001	-	-
Dialysis	2.48 (2.20-2.80)	<0.001	1.07 (0.91-1.3)	0.417
Cancer	1.63 (1.31-2.03)	<0.001	-	-
Steroid use	1.61 (1.43-1.80)	<0.001	-	-
Bleeding disorder	1.63 (1.53-1.74)	<0.001	1.34 (1.24-1.45)	<0.001
Blood transfusion	2.83 (2.51-3.19)	<0.001	1.24 (1.06-1.44)	0.006
Wound infections	2.05 (1.76-2.39)	<0.001	-	-
Systemic inflammation				
None	Reference	-	-	-
SIRS	3.03 (2.72-3.37)	<0.001	1.66 (1.47-1.89)	<0.001
Sepsis/shock	8.79 (7.21-10.71)	<0.001	3.83 (3.04-4.82)	<0.001
Perioperative and operative characteristics				
Emergency case	2.41 (2.24-2.58)	<0.001	-	-
Ventilator dependent	11.98 (9.77-14.68)	<0.001	4.17 (3.25-5.36)	<0.001
Baseline laboratory markers (preoperative)				
Creatinine (mg/dL)				
<1.5	Reference	-	-	-
>1.5	2.26 (2.12-2.42)	<0.001	1.59 (1.46-1.74)	<0.001
Albumin (g/dL)				
>3.5	Reference	-	-	-
≤3.5	2.10 (1.99-2.21)	<0.001	1.41 (1.33-1.50)	<0.001
WBC (10^9^/L)				
4 to 10	Reference	-	-	-
<4 or >10	1.91 (1.81-2.02)	<0.001	1.38 (1.29-1.48)	<0.001
Hematocrit (%)				
≥40	1.62 (1.54-1.70)	<0.001	1.11 (1.05-1.19)	<0.001
<40	Reference	-	-	-

Sensitivity analysis

Because perioperative albumin was missing in 20% of patients, we performed a sensitivity analysis with the best- and worst-case scenarios. The best-case scenario assumed all missing data patients had perioperative albumin greater than 3.5 g/dL. The worst-case scenario assumed all missing data patients had perioperative albumin less than or equal to 3.5 g/dL. Re-analyzing the data under both scenarios indicated that our point estimate was reasonably robust to missing data (best-case scenario OR 1.42, 95% CI 1.34-1.51; worst-case scenario OR 1.17, 95% CI 1.11-1.23).

## Discussion

We found that, compared to independent patients, those with dependent functional status had 21% higher odds of developing the primary composite outcome of major postoperative events, even after adjusting for variables such as age, gender, comorbidities (CHF, dialysis, bleeding disorder, blood transfusion, SIRS, sepsis/shock), perioperative ventilator dependence, and laboratory markers (creatinine, albumin, WBC, and Hct).

Several other studies found similar associations, albeit in non-cardiac surgery such as vascular and bariatric. For instance, one paper found that patients who were functionally dependent by NSQIP classification had a three times higher likelihood of 30-day mortality after endovascular aortic repair compared to partially dependent or independent patients, after multivariable adjustment for demographics, comorbidities, and operative risk [8]. Another paper investigating heart transplant recipients found that pre-transplant functional status was a good predictor of post-transplant survival, with better functional status (based on Karnofsky performance scores) having higher rates of 30-day and one-year survival [11]. In terms of other complications, such as unplanned reintubation, sepsis, or cardiac arrest, the literature is also consistent in documenting that patients who experienced these events were much more likely to be partially or totally dependent by NSQIP classification for ADLs prior to surgery [12-14].

There are several proposed explanations as to why preoperative dependent functional status would lead to worse patient outcomes after surgery. Patients with decreased physical activity preoperatively have a higher risk of a postoperative complicated recovery, which includes occurrences of reoperation, deep wound infections, renal failure, stroke, postoperative ventilation, mortality, and longer length of stay [15]. Furthermore, undergoing surgery is a major stressor on the body, with elevation of pro-inflammatory mediators, immune dysregulation, and increased muscle proteolysis both from healing as well as decreased activity immediately post-surgery [16]. Though most patients are able to regain function after a period of recovery, patients who are already in poor functional status prior to surgery may not have an adequate surgical stress response, leading to additional postoperative complications or comorbidities [17]. 

It is important to note that functional status is a global measurement of health and therefore has a complex relationship with many of the other variables in our study. For example, we found that among the dependent functional status group, there were greater proportions of patients who were elderly (≥80), female, minority race/ethnicities, underweight/obese, and comorbid; this aligns with what previous studies have found regarding factors associated with greater postoperative complications [18,19]. There was also a greater prevalence of abnormal laboratory markers (creatinine, albumin, WBC, and Hct) among the dependent patients. This may be associated with the overall higher comorbidity burden in the dependent patients (for example, a greater prevalence of underweight status in the dependent group may be associated with lower albumin values due to malnutrition) [20]. Literature is limited on the relationship between comorbidities and functional status, but recent papers actually suggest that comorbidity and functional status are relatively independent and play different roles in determining patient preoperative status [21,22]. Indeed, in our study, the association between functional status and postoperative complications persisted even after extensive adjustment for comorbidities. 

We found a higher prevalence of overweight status in the independent functional group and a lower incidence of the primary outcome in patients who were overweight compared to patients with normal BMI. Though some papers have suggested that being overweight does impair functional status and increases the risk of operative complications [23-24], there is evidence that being overweight or even moderately obese may have no significant impact on patient outcomes or may even be associated with a lower risk compared to patients with normal weight [25,26].

Incidentally, we also found that being ventilator dependent prior to operation as well as having sepsis and/or septic shock 48 hours prior to operation significantly increased odds of developing postoperative complications. This can be explained by the fact that both are associated with more severe health conditions, and these patients often require emergency surgery due to a significantly higher risk of morbidity and mortality without intervention [27,28]. Both variables had increased prevalence in the dependent vs. independent functional status groups, and are important confounders in the relationship between functional status and postoperative complications.

There is an increasing body of evidence supporting the efficacy of prehabilitation programs in improving outcomes for dependent patients undergoing cardiac surgery. A recent study found that patients who engaged in exercise-based prehabilitation, with a minimum cumulative duration of 90 minutes per week and a minimum program length of two weeks, experienced shorter lengths of hospital stay (mean difference -1.00 day, 95% CI -1.78 to -0.23 days) and lower risk of postoperative atrial fibrillation (risk ratio 0.34, 95% CI 0.14-0.83) compared with controls who received standard care [29]. These findings highlight the impact of implementing prehabilitation as a part of a comprehensive preoperative care protocol in this patient population, which can ultimately contribute to improved overall outcomes and quality of life.

Limitations

Our study results need to be interpreted in light of its inherent limitations. Due to its retrospective design, causality cannot be established. Despite adjusting for a variety of covariates, we cannot exclude the possibility of residual confounding. Additionally, other outcomes of interest, such as perioperative arrhythmias or valvular diseases, and relevant factors such as pain scores, neurological status, and potassium levels, were not available in the NSQIP data. Furthermore, our analysis was constrained by a high percentage (20-30%) of missing values in dyspnea and race. Given the low prevalence of totally dependent status, we combined partially and totally dependent patient groups.

Regarding external validity, one major limitation is that NSQIP data are collected only from participating hospitals, thus the sample may not be fully representative of the entire population undergoing cardiac surgery. Additionally, hospitals with better outcomes may be more likely to participate, potentially introducing selection bias.

## Conclusions

Our findings emphasize the importance of considering a patient's functional status before they undergo cardiac surgery. Healthcare professionals may be able to provide more tailored medical care by seeking alternative treatment options. We found that participants with partial or total dependent status exhibited higher odds of complications than independent participants, both before and after adjusting for confounders. The most common complications observed were unplanned reoperation, ventilator dependency over 48 hours, pneumonia, and unplanned intubation.

Future research could explore the development and clinical testing of a predictive tool including functional status. This tool could help clinicians identify high-risk patients and facilitate timely interventions to improve patient outcomes. Additionally, further investigations could assess the effectiveness of prehabilitation programs in enhancing the functional capacity of dependent patients before cardiac surgery, as well as their potential impact on surgical outcomes.
